# A *cis*-Acting Diversification Activator Both Necessary and Sufficient for AID-Mediated Hypermutation

**DOI:** 10.1371/journal.pgen.1000332

**Published:** 2009-01-09

**Authors:** Artem Blagodatski, Vera Batrak, Sabine Schmidl, Ulrike Schoetz, Randolph B. Caldwell, Hiroshi Arakawa, Jean-Marie Buerstedde

**Affiliations:** Institute for Molecular Radiobiology, Helmholtz Center Munich, Neuherberg, Germany; The Jackson Laboratory, United States of America

## Abstract

Hypermutation of the *immunoglobulin* (*Ig*) genes requires Activation Induced cytidine Deaminase (AID) and transcription, but it remains unclear why other transcribed genes of B cells do not mutate. We describe a reporter transgene crippled by hypermutation when inserted into or near the *Ig light chain* (*IgL*) locus of the DT40 B cell line yet stably expressed when inserted into other chromosomal positions. Step-wise deletions of the *IgL* locus revealed that a sequence extending for 9.8 kilobases downstream of the *IgL* transcription start site confers the hypermutation activity. This sequence, named *DIVAC* for *diversification activator*, efficiently activates hypermutation when inserted at *non-Ig* loci. The results significantly extend previously reported findings on AID-mediated gene diversification. They show by both deletion and insertion analyses that *cis*-acting sequences predispose neighboring transcription units to hypermutation.

## Author Summary

It remains an open question how AID-mediated gene diversification is targeted to the immunoglobulin loci. Here we define a *cis*-acting sequence, named *DIVAC* for diversification activator, which is required for hypermutation of the *Ig* light chain gene and sufficient to activate hypermutation at various *non-Ig* loci in the DT40 B cell line. *DIVAC* is composed of multiple interacting sequences and able to work over considerable distances both upstream and downstream of its target gene. This work provides the first conclusive evidence that AID-mediated gene diversification is targeted to the *Ig* loci by *cis*-acting sequences. The conservation of AID-mediated *Ig* gene diversification during vertebrate evolution suggests that *DIVAC*s also play a role in gene conversion, hypermutation, and switch recombination in mammalian B cells. The findings should be of general interest not only for molecular immunology and the pathogenesis of B cell lymphomas but also the whole field of biology as a unique example of how locus-specific gene diversification is controlled. The described experimental system offers unique advantages to further clarify the molecular mechanism of *DIVAC*.

## Introduction

Vertebrate B cells are able to diversify their rearranged *immunoglobulin* (*Ig*) genes by hypermutation, gene conversion and class switch recombination. All three phenomena require expression of Activation Induced cytidine Deaminase (AID, NC_006088) [1]–[3] which most likely initiates *Ig* gene diversification by deaminating cytidines within the mutating and recombining sequences [4],[5]. A further requisite for hypermutation and switch recombination is the transcription of the *Ig* genes and the switch regions respectively [6],[7].

Sequence analysis of transcribed *non-Ig* genes from AID expressing B cells revealed either no or only a low number of mutations compared to *Ig* genes [8]. A recent study of a large number of expressed genes in B cells found a significantly higher number of mutations in wild-type mice than in *AID* knock-out mice [9]. However, the mutation rates for the *non-Ig* genes in AID expressing B cells were still orders of magnitude lower than for the *Ig* genes. To explain this difference between *Ig* and *non-Ig* genes it has been postulated that *cis*-acting sequences in the *Ig* loci activate hypermutation possibly by recruiting AID. However, intense efforts did not succeed to unambiguously define these sequences for the murine and human *Ig* loci [10]. Whereas studies using chimeric reporter genes in transgenic mice indicated that certain *Ig* enhancers and their surrounding sequences conferred hypermutation activity [11]–[14], deletion of *Igκ* enhancers in knock-out mice did not prevent hypermutation of the *Igκ* gene (CAA36032) [15],[16]. At least one murine B cell line [17] and AID expressing fibroblasts [18] mutated transcribed transgenes in the absence of nearby *Ig* locus sequences, further confounding the issue of whether *cis*-acting regulatory sequences are needed for hypermutation.

The chicken B cell line DT40 diversifies its rearranged *Ig light chain* (*IgL*) gene by gene conversion in the presence of nearby *pseudo V* (*ψV*) genes [2] and by hypermutation, if the *ψV* genes are deleted [19]. Both activities strictly depend on the expression of AID. Consistent with the idea that the absence of homologous gene conversion donors leads to hypermutation, a *Green Fluorescent Protein* (GFP, AAB08058) transgene is rapidly diversified by mutations when inserted into the rearranged *IgL* locus [20]. The hypermutation activity of DT40 appeared however to be limited to the *IgL* locus, because no mutations were found in the highly transcribed *Elongation Factor 1 alpha* gene (NP_989488) [19]. This was confirmed by a recent study showing that neither the *VpreB3* (NC_006102) nor the *Carbonic Anhydrase* (XP_415218) gene, immediate upstream and downstream neighbors of the *IgL* locus respectively, showed sequence heterogeneity in DT40 [21].

DT40 has been proposed as a model to study the mechanism of *Ig* hypermutation [19]. Compared to mice and humans, the chicken *IgL* locus including the *ψV* genes is compact spanning only 30 kb. Furthermore, targeted integration of transfected DNA constructs in DT40 allows the introduction of deletions and insertions at defined chromosomal positions. Encouraged by these advantages we decided to search for the elusive *cis*-acting hypermutation control sequence in the *IgL* locus of DT40.

## Results

### A Hypermutation Reporter Based on GFP Expression

**Figure 1:**
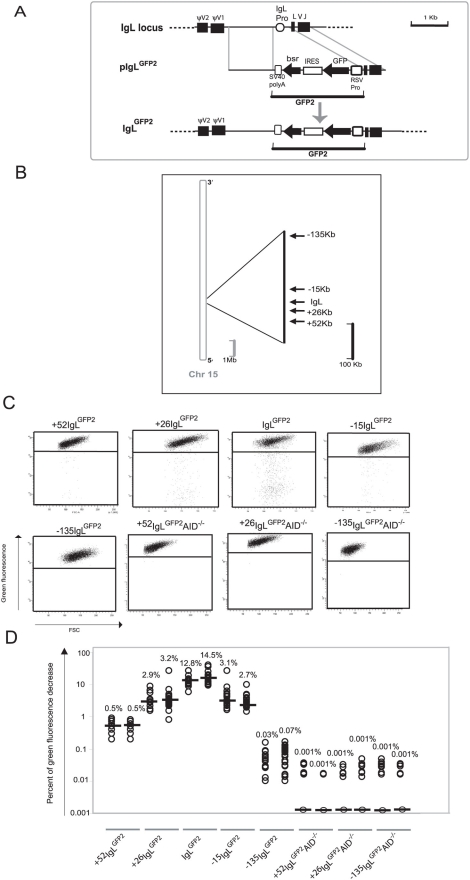
Hypermutation of the *GFP2* reporter at various distances from the rearranged *IgL* locus. (A) A physical map of the rearranged *IgL* locus, a targeting construct including the *GFP2* reporter and the *IgL* locus after targeted insertion of the *GFP2* reporter. (B) Locations of *GFP2* insertion relative to the *IgL* locus on chromosome 15. The reference point is the insertion site of *GFP2* in the IgL^GFP2^ transfectant. (C) FACS analysis of primary transfectants having integrated the transfected construct targeted. (D) Fluctuation analysis of subclones. Each dot represents the analysis of a single subclone and the median of all subclones from the same primary transfectant is indicated by a bar.

We have previously demonstrated that a *GFP* transgene in DT40 rapidly accumulates mutations, when integrated at the position of the promoter of the rearranged *IgL* locus [20]. The hypermutation activity depended on AID expression and could be visualized by the appearance of cells displaying decreased green fluorescence due to detrimental *GFP* mutations. To exploit this phenomenon, we designed a new expression cassette named *GFP2* which consisted of the strong *RSV* promoter followed by the *GFP* coding region, an *internal ribosome entry site* (*IRES*), the *blasticidin resistance* gene (P19997) and the *SV40* polyadenylation signal. *GFP2* was incorporated into the targeting construct *pIgL^GFP2^* (Figure 1A) in the opposite transcriptional orientation of the *IgL* gene to minimize interference between transcriptional and post-transcriptional regulation of the *GFP2* transgene and the *IgL* gene.

Transfection of *pIgL^GFP2^* into the conditionally AID expressing clone AID^R2^ yielded a number of transfectants named IgL^GFP2^ in which targeted integration had substituted the *IgL* promoter by the *GFP2* transgene. Fluorescence activated cell sorting (FACS) analysis of subclones from two independent primary transfectants revealed median values of 12.8% and 14.5% decreased green fluorescence two weeks after subcloning (Figure 1C and 1D). The result confirms our previous study indicating that the *GFP2* transgene is mutated at high rate within the rearranged *IgL* locus and that cell populations with decreased green fluorescence can be used to quantify this hypermutation activity.

### Hypermutation in the Vicinity of the *IgL* Locus

Targeted integration was used to insert the *GFP2* reporter at various distances from the *IgL* locus into chromosome 15 [22] (Figure 1B and Figure S1A). FACS analysis of primary transfectants (Figure 1C and Table S1) and their subclones (Figure 1D) revealed that the medians of decreased green fluorescence fell to about 3% at the +26 kb and the −15 kb positions, to 0.5% at the +52 kb position and to about 0.05% at the −135 kb position. The medians of decreased green fluorescence were only around 0.001% at the +52, +26 and −135 kb positions in the absence of AID (Figure 1C and 1D and Table S1), indicating that the decreased green fluorescence was dependent on AID expression.

Although we did not determine for the *GFP2* insertions outside the *IgL* locus, whether the rearranged or the unrearranged allele was targeted, the results were representative for a large number of independent primary transfectants (Table S1). Thus, hypermutation of the *GFP2* reporter was detectable at insertions up to 52 kb away from the *IgL* locus, but mutations declined with increasing distance and were barely detectable at the −135 kb position.

Since surrounding sequences were unlikely to influence the post-transcriptional processing and translation of *GFP2* transcripts, *GFP2* transcription should be reflected by the green fluorescence of the cells independent of the transgene insertion site. Even in the case of mutating transgenes, *GFP2* transcription levels could be deduced from the average green fluorescence of the major cell populations which most likely expressed the un-mutated *GFP* sequence. As seen by FACS analysis, the average green fluorescence of the major cell populations varied slightly among the primary transfectants (Figure 1C) most likely reflecting chromosomal position effects. However, the transfectants +52IgL^GFP2^ and IgL^GFP2^ differed more than 20 fold in their median fluorescence decreases despite similar green fluorescence of their major cell populations. This strongly suggested that the hypermutation differences among the transfectants reflected the distance of the *GFP2* insertion sites to the *IgL* locus and not variation in *GFP2* transcription.

### Identification of a Diversification Activator

The results could be explained by the presence of a *cis*-acting sequence which activated hypermutation in a distance dependent manner. We have named this putative regulatory sequence Diversification Activator *(DIVAC)* and attempted to map it by combining insertions of the *GFP2* reporter with deletions of the *IgL* locus.

**Figure 2:**
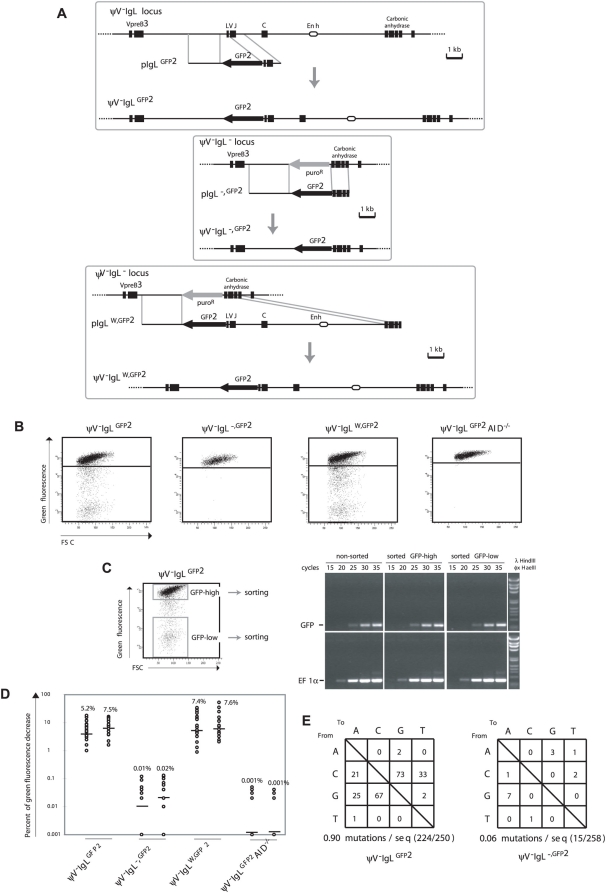
*GFP2* hypermutation after deletion and reconstitution of the rearranged *IgL* locus. (A) Design of deletions and insertions in the rearranged *IgL* locus using *GFP2* targeting constructs. Upper part: Physical maps of the rearranged *IgL* locus after the deletion of the *ψV* locus, a targeting construct including the *GFP2* reporter and the locus after targeted integration. Middle part: Physical maps of the deleted rearranged *IgL* locus, a targeting construct including the *GFP2* reporter and the *GFP2* insertion site after targeted integration. Lower part: Physical maps of the deleted rearranged *IgL* locus, a targeting construct including the *GFP2* reporter together with the ‘*W*’ fragment and the locus after targeted integration. (B) FACS analysis of primary transfectants. (C) Sorting of GFP-high and GFP-low cells from a ψV^−^IgL^GFP2^ primary transfectant, and semi-quantitative RT-PCR of *GFP* and *EF1α* messages from sorted and non-sorted cells. (D) Fluctuation analysis of subclones. (E) The frequencies of particular nucleotide substitutions within the *GFP* open reading frame of *GFP2*.

To address the role of the *ψV* part of the *IgL* locus, a *GFP2* construct (Figure 2A, upper part) was transfected into the clone ψV^−^AID^R1^ [20] in which the entire 20 kb containing the *ψV* genes had been deleted. The transfectants ψV^−^IgL^GFP2^ expressed the *GFP2* reporter at the position of the *IgL* promoter in the absence of the *ψV* locus.

FACS analysis of ψV^−^IgL^GFP2^ primary transfectants showed sizable populations of cells showing decreased green fluorescence (Figure 2B) indicating that the GFP gene is diversified by hypermutation. To rule out that the decrease of green fluorescence is caused by gene silencing, the populations of high (GFP-high) and low (GFP-low) green fluorescence were sorted from one of the ψV^−^IgL^GFP2^ primary transfectants (Figure 2C, left), and *GFP* mRNA levels was analyzed by semi-quantitative reverse transcription polymerase chain reaction (RT-PCR), (Figure 2C, right). RT-PCR of the *EF1α* mRNA served as a control. Although GFP-low cells showed on average more than 100-fold lower green fluorescence than GFP-high cells, the levels of *GFP* mRNA were comparable between sorted GFP-low, GFP-high and non-sorted cells, confirming that the decrease of green fluorescence is not due to silencing of GFP gene expression.

FACS analysis of ψV^−^IgL^GFP2^ subclones revealed medians of 5.2% and 7.5% decreased green fluorescence (Figure 2D), only one fold lower than the medians of the *ψV* positive IgL^GFP2^ subclones. As the difference between IgL^GFP2^ and ψV^−^IgL^GFP2^ subclones could be due to fluctuation effects or different AID expression levels in the AID^R2^ and ψV^−^AID^R1^ precursors, the *ψV* locus seems to exert little, if any stimulation on the hypermutation activity of the *GFP2* reporter.

ψV^−^IgL^GFP2^ still contained a 9.8 kb fragment of the rearranged *IgL* locus extending from the *IgL* transcription start site until the 3′ end of the carbonic anhydrase gene and referred to in the following as fragment ‘*W*’. To test the relevance of this fragment, a *GFP2* construct was transfected into the clone ψV^−^IgL^−^ in which the entire rearranged *IgL* locus had been replaced by the *puromycin resistance* gene (P42670). The resulting transfectants ψV^−^IgL^−,GFP2^ had the *puromycin resistance* gene replaced by the *GFP2* reporter at the position of the deleted *IgL* locus (Figure 2A, middle part, and Figure 2B). Subclones of ψV^−^IgL^−,GFP2^ showed medians of only 0.01% and 0.02% decreased green fluorescence (Figure 2D), more than 100 fold lower than the medians of ψV^−^IgL^GFP2^ subclones. This indicated that the ‘*W*’ fragment, absent in ψV^−^IgL^−,GFP2^ but present in ψV^−^IgL^GFP2^, was required for hypermutation of the *GFP2* transgene. ψV^−^IgL^−^ cells were then transfected by a construct including the *GFP2* transgene and the ‘*W*’ fragment (Figure 2A, lower part). Subclones of the transfectants ψV^−^IgL^W,GFP2^ showed median green fluorescence decreases similar to the medians of ψV^−^IgL^GFP2^ subclones (Figure 2D). Thus, the ‘W’ fragment efficiently activates hypermutation after reinsertion into the *IgL* locus as expected for a true *DIVAC* sequence.

Controls confirmed that the appearance of cells with decreased green fluorescence reflected hypermutation in the *GFP2* gene. As expected, the decrease of green fluorescence in ψV^−^IgL^GFP2^ cultures depended on AID, because subclones of the AID negative transfectant ψV^−^IgL^GFP2^AID^−/−^ showed only very low medians of 0.001% decreased green fluorescence (Figure 2D). Furthermore, 723 bp of the *GFP* open reading frame amplified from ψV^−^IgL^GFP2^ cells six weeks after subcloning showed an average of 0.9 nucleotide substitutions per sequence (Figure 2E). As the doubling time of the DT40 cell line is about 10 hours, the mutation rate of the *GFP* gene of ψV^−^IgL^GFP2^ was calculated to be 1.3×10^−5^ mutation/bp/generation, which was similar to the mutation rate of the human hypermutating RAMOS cell line (2.2×10^−5^ mutation/bp/generation) [23],[24]. The most prevalent mutations were *C* to *G* and *G* to *C* transversions as previously observed for hypermutation of the *IgL VJ* segments from *ψV* deleted DT40 clones [19]. In contrast, only a very low number of nucleotide substitutions, most likely reflecting polymerase chain reaction (PCR) artifacts, were found in the *GFP* gene of ψV^−^IgL^−,GFP2^ cells (Figure 2E).

### Fine Mapping of *DIVAC*

**Figure 3:**
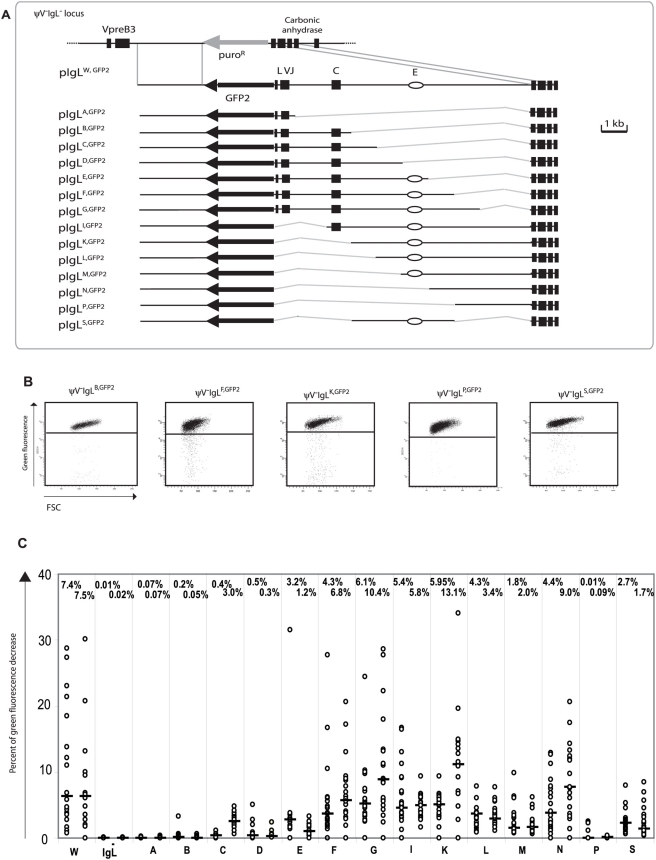
Analysis of the ‘*W*’ fragment deletion series. (A) A physical map of the deleted *IgL* locus and the aligned targeting constructs leading to the insertion of the *GFP2* reporter together with parts of the ‘*W*’ fragment. (B) FACS analysis of representative primary transfectants. (C) Fluctuation analysis of subclones. Only the letter of the reconstituted fragment and not the full name of the transfectants is indicated for clarity.

A new series of targeting constructs was transfected into ψV^−^IgL^−^ to characterize the ‘*W*’ fragment by step-wise deletions (Figure 3A). FACS analysis of subclones from the different transfectants showed a variable but progressive loss of hypermutation activity when the ‘*W*’ fragment was shortened from either end (Figure 3C). The 4 kb ‘*S*’ fragment in the middle of the ‘*W*’ fragment, which included the previously identified *IgL* enhancer [25], still produced median green fluorescence decreases of 2.7% and 1.7%. In contrast, the upstream ‘*B*’ and the downstream ‘*P*’ fragments on their own produced median green fluorescence decreases of 0.13% and 0.05% respectively, which are low in absolute terms, but clearly above the medians of ψV^−^IgL^−,GFP2^. If either one of these fragments was combined with the ‘*S*’ fragment in the ‘*F*’ and ‘*K*’ fragments respectively, the median decreases of green fluorescence were elevated about 3 times. This suggested that the *DIVAC* of the chicken *IgL* locus consisted of a central core region and partially redundant flanking regions which contributed to the overall activity. Clearly, more detailed analysis is needed to define the location, the nature and the configuration of the active motifs within the *IgL DIVAC*.

The average green fluorescence in the main population of ψV^−^IgL^W,GFP2^ was increased compared to ψV^−^IgL^−,GFP2^ (Figure 2B) perhaps due to the additional stimulation of the *RSV* promoter in *GFP2* by the *IgL* enhancer of the ‘*W*’ fragment. However, the relatively small decrease of *GFP2* transcription seen in ψV^−^IgL^−,GFP2^ was unlikely to be responsible for the more than 300 fold reduction of hypermutation. Analysis of the ‘*W*’ fragment deletions also strongly argued against the possibility that differences in hypermutation were caused by alterations of *GFP2* transcription since primary transfectants of all fragments shown in Figure 3B showed similar *GFP* transcription levels. Similar levels of steady-state *GFP2* transcripts were confirmed by RT-PCR (Figure S2).

### Hypermutation at *non-Ig* Loci

**Figure 4:**
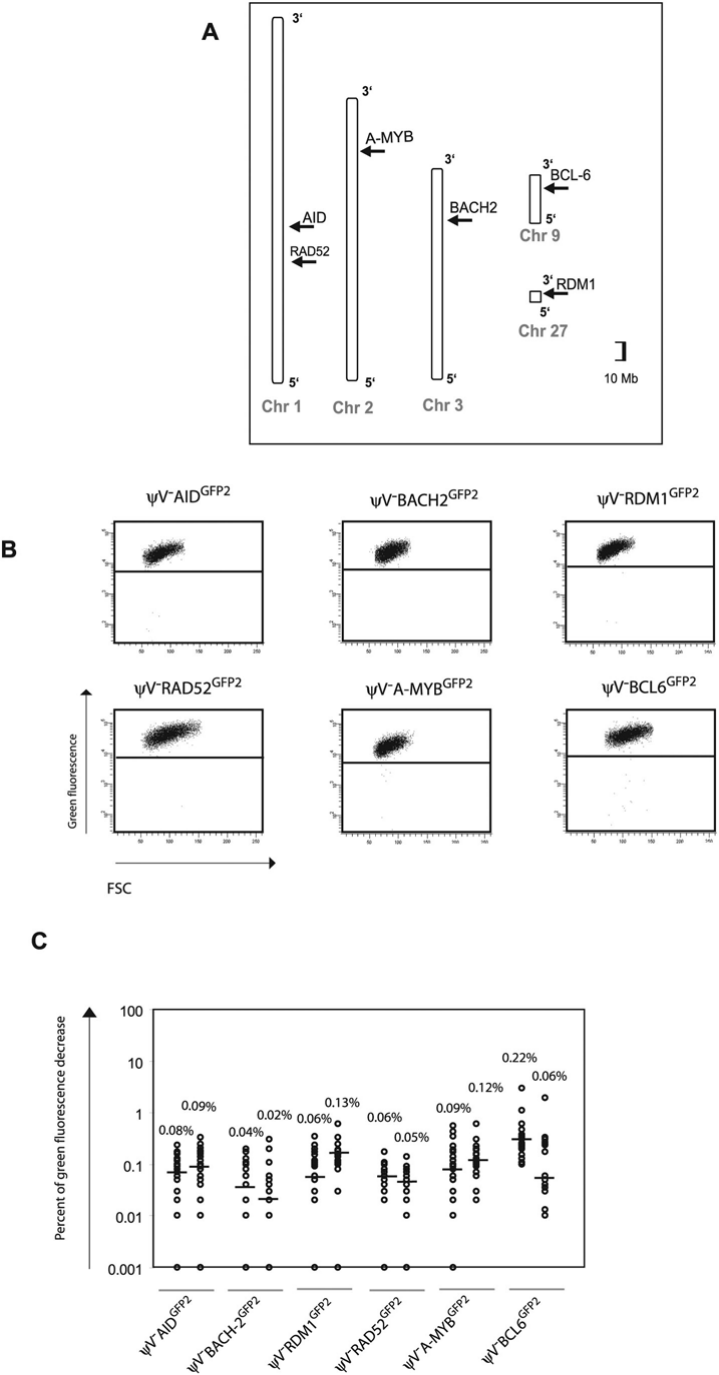
Insertions of the *GFP2* reporter into *non-Ig* loci. (A) Chromosomal locations of the *GFP2* insertions. (B) FACS analysis of primary transfectants. (C) Fluctuation analysis of subclones.

**Figure 5:**
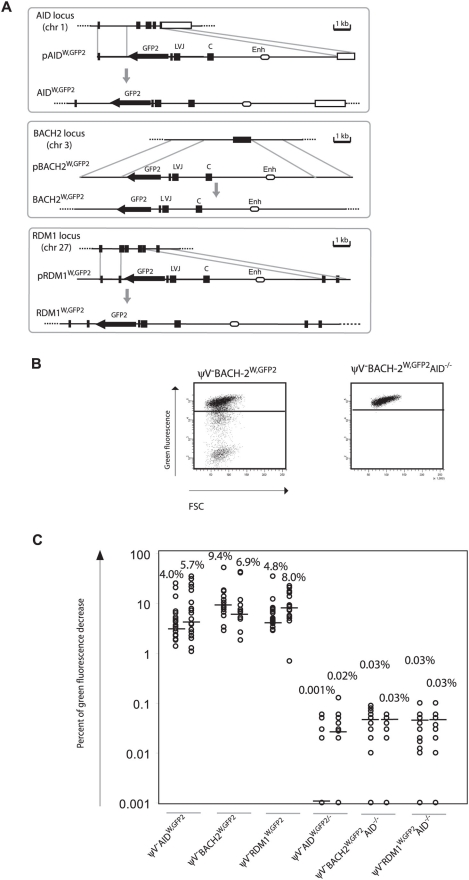
Hypermutation of the *GFP2* reporter at *non-Ig* loci in the presence of the ‘*W*’ fragment. (A) Physical maps of the *non-Ig* loci, the targeting constructs including the *GFP2* reporter together with the ‘*W*’ fragment and the loci after targeted insertion of the *GFP2* reporter. (B) FACS analysis of primary *AID* positive and *AID* negative transfectants. (C) Fluctuation analysis of subclones.

To confirm that the *GFP2* reporter on its own is stably expressed at *non-Ig* loci, six loci on five different chromosomes [22] were targeted by transfection of *GFP2* constructs into ψV^−^AID^R1^ (Figure 4A and Figure S1B). Neither the primary transfectants (Figure 4B and Table S1B) nor their subclones (Figure 4C) showed high percentages of decreased green fluorescence. Depending on the experiment and the insertion site, the medians of the subclones ranged from 0.02% to 0.22% indicating that the mutation rates of the *GFP2* reporter at the chosen loci were 50 to 500 fold lower than at the *IgL* locus. However, these medians were about 2–10 fold higher than the medians of various subclones from AID negative transfectants (Figures 2D and 5C) confirming a slight increase in the background mutation rates in AID expressing B cells [9].

*GFP2* was then inserted together with the ‘*W*’ fragment into the respective *AID*, *BACH2* (NC_006090) and *RDM1* (BAC02561) loci of ψV^−^AID^R1^ (Figure 5A and 5B). Subclones of the transfectants ψV^−^AID^W,GFP2^, ψV^−^BACH2^W,GFP2^ and ψV^−^RDM1^W,GFP2^ showed high median decreases of green fluorescence between 4.0% and 9.4% (Figure 5C) similar to the medians for ψV^−^IgL^GFP2^ and ψV^−^IgL^W,GFP2^ subclones. *GFP2* hypermutation was AID dependent, since subclones of the *AID* negative transfectants ψV^−^AID^W,GFP2/−^, ψV^−^BACH2^W,GFP2/−^AID^−/−^ and ψV^−^RDM1^W,GFP2/−^AID^−/−^ showed very low medians of decreased green fluorescence in the range of 0.001% to 0.03%. These results demonstrated that the ‘*W*’ fragment was able to activate *AID* mediated hypermutation at loci which otherwise did not support hypermutation.

## Discussion

We have identified a *cis*-acting sequence that is needed for hypermutation at the chicken *IgL* locus and able to activate hypermutation at other loci upon insertion. The 9.8 kb sequence, named *DIVAC* for diversification activator, extends from the *IgL* transcription start site towards the next downstream gene. *DIVAC* seems to be composed of multiple interacting regions. Whereas a 4 kb core sequence which includes the known *IgL* enhancer activates hypermutation more than 100 fold above background level, the flanking regions possess less activity on their own, but stimulate hypermutation when combined with the core. Surprisingly, *DIVAC* can act on either side of the hypermutation reporter and over long distances.

Given the conservation of AID mediated *Ig* gene diversification during vertebrate evolution, the identification of the chicken *IgL DIVAC* should also be of relevance for mammals. Searches for *cis*-acting hypermutation regulatory sequences in transgenic mice showed that regions surrounding the *Ig* enhancers conferred hypermutation activity [11]–[14]. Intriguingly, the location and functional characteristics of these regions appear to be similar to the core of the chicken *IgL DIVAC*. In hindsight, the difficulty to unambiguously prove the existence of hypermutation activator sequences may relate to the large size of the murine *Ig* loci and the fact that *DIVAC*s seem to be composed of multiple interacting regions. As each of the murine *Ig* loci possesses at least two enhancers at different positions, murine *DIVAC*s may be composed of multiple discontinuous sequences.

Bursal B cells and DT40 in the presence of nearby *ψV* genes diversify their rearranged *IgL* loci by gene conversion, suggesting that one of the physiological roles of the *IgL DIVAC* is activation of gene conversion. This is supported by a recent publication showing that a deletion of the rearranged *IgL* locus downstream of the *C* region stopped *IgL* gene diversification in *ψV* positive DT40 [26]. It seems also likely that *DIVACs* play a role for switch recombination which is accompanied by hypermutation of the recombining switch regions [27]. Possibly a dedicated *DIVAC* near the switch regions activates switch recombination. As the chicken *IgL DIVAC* can activate hypermutation in both directions over large distances, it is also conceivable that a single *DIVAC* in the heavy chain loci regulates both hypermutation and switch recombination.

The mechanism of how a *cis*-regulatory sequence can activate hypermutation in neighboring transcription units remains speculative. Intriguingly, the chicken *IgL DIVAC* not only includes the *IgL* enhancer, but also seems to act like an enhancer by activating hypermutation over long distances in upstream or downstream target genes. A plausible hypothesis may be that *DIVAC* promotes the formation of protein complexes which first bind AID and then hand it over to the neighboring transcription initiation complex. Candidates for proteins involved in building such an AID docking station would be DNA binding factors which recognize sequence motifs within *DIVAC*. The described experimental system offers unique advantages to test this hypothesis.

## Materials and Methods

### Targeting Constructs

The *GFP2* construct was made by combining the *RSV* promoter-*GFP* open reading frame of *pHypermut2* [20] with a PCR amplicon including an *IRES* [19], the *blasticidin resistance* gene and the *SV40* polyadenylation signal [28]. PCR was performed using the primers described in the Table S2. *GFP2* was flanked by unique *BamHI* restriction sites for easy cloning into the targeting vectors.

All targeting constructs except the ones belonging to the series of ‘*W*’ fragment deletions and reconstitutions were made by cloning the arms sequences into *pBluescriptKS+* (Stratagene, CA) and then inserting *GFP2* either into unique *BamHI* or *BglII* sites as shown in Figure 5A and Figure S1. Targeting of *AID* [28] and *RDM1* [29] have been previously described. PCR amplifications of all target arms were performed using the Expand long template PCR System (Roche, Switzerland), DT40 genomic DNA as template and primers as described in the Table S2.

Since the ‘*W*’ fragment was difficult to amplify as a single sequence, it was sequentially cloned by combining upstream and downstream PCR amplicons with a 2.2 kb *AvrII*/*SpeI* restriction fragment excised from the rearranged *IgL* targeting construct ‘*Construct R*’ [30]. The sequence of the *AvrII*/*SpeI* restriction fragment is *A*/*T* rich and localized between the *J* segment and the *C* region. The assembled ‘*W*’ fragment of 9784 nucleotides was sequenced and deposited into Genbank under the accession number FJ482234. It starts at position −7 relative to the first base of the *IgL* start codon and corresponds to the chicken genome coordinates chr15:8165070–8176699 but lacks the *VJ* intervening sequence.

Constructs belonging to ‘*W*’ fragment deletion series were made by cloning *GFP2* between the target arms and then inserting the ‘*W*’ fragment or parts thereof into unique *NheI*/*SpeI* sites. A *BamHI* fragment containing *GFP2* and the ‘*W*’ fragment was incorporated into the *AID*, *BACH2* and *RDM1* targeting vectors to test the activity of the ‘*W*’ fragment in *non-Ig* loci.

### Cell Culture

Cells were cultured in chicken medium (RPMI-1640 or DMEM/F-12 with 10% fetal bovine serum, 1% chicken serum, 2 mM L-glutamine, 0.1 µM β-mercaptoethanol and penicillin/streptomycin) at 41°C with 5% CO2. Transfections were performed by electroporation, using 40 µg of linearized plasmid DNA with a Gene Pulser Xcell (BIO-RAD) at 25 µF and 700 V. Stable transfectants were selected by culturing in 15 µg/ml of blasticidin. Transfectants having integrated the transgenic constructs by targeted integration were identified by PCR using an inside primer from the *SV40* polyadenylation signal sequence of *GFP2* together with a primer derived from the sequence outside the target arm (Table S2). In case of insertions into the *IgL* locus, targeted integration into the rearranged allele was verified by amplifying the *VJ* intervening sequence of the unrearranged locus. The *AID* reconstituted clone AID^R2^ was generated from the *AID* deleted clone AID^−/−^ [2] by transfection of a construct which targeted an *AID* cDNA expression cassette into one of the deleted *AID* loci. The AID negative transfectants were produced by transfecting AID^−/−^ and ψV^−^AID^−/−^ [19], respectively.

### Flow Cytometry

The phenotype of each mutation was determined by FACS analysis of at least two independent targeted transfectants and twenty-four subclones of each. The primary transfectants were analyzed by FACS about three weeks after transfection and the subclones two weeks after subcloning. As the green fluorescence levels in the main populations varied slightly among the transfectants, the gates to separate the main population of green fluorescent cells from cells showing decreased or lost green fluorescence were adapted accordingly. At least 5000 events falling into the live cell gate were collected for each primary transfectant or subclone. Subclones in which more than 50% of the live cell events fell into the gates for decreased or lost green fluorescence were excluded from the analysis as they might represent the expansion of a precursor cell already expressing a mutated *GFP2* transgene at the time of subcloning.

### *GFP* Gene Sequencing

To minimize PCR-introduced mutations, *Pfu* Ultra hotstart polymerase (Stratagene) was used for the amplifications of the *GFP* open reading frames prior to sequencing. Sequencing and sequence analysis were performed as previously described [19].

### RT-PCR

RT-PCR was performed as previously described [2]. Primer pairs used for amplification of the *GFP* and *elongation factor 1α* transcripts are shown in Table S2.

## Supporting Information

Figure S1 (A) Targeting strategy of the *GFP2* reporter into four different loci on chromosome 15. (B) Targeting strategy of the *GFP2* reporter into the *A-MYB*, *RAD52*, *BACH2*, and *BCL6* loci. The targeting strategies used for the insertions into the *AID* and the *RMD1* loci were described previously [2],[29].
(1.87 MB EPS)

Figure S2 Comparison of GFP gene expression levels analyzed by semi-quantitative RT-PCR of primary transfectants belonging to the stepwise deletions series of the ‘*W*’ fragment.
(2.27 MB EPS)

Table S1 Green fluorescence decrease in individual primary transfectants. (A) GFP2 reporter in the vicinity of the *IgL* locus. (B) GFP2 reporter in *non-Ig* loci.
(0.11 MB XLS)

Table S2 List of primers.
(0.12 MB DOC)
