# Supplementary material for: A cis-Acting Diversification Activator Both Necessary and Sufficient for AID-Mediated Hypermutation
Source: PLoS Genet. 2009 Jan 9;5(1):e1000332. doi: 10.1371/journal.pgen.1000332 (PMC2607555; doi:10.1371/journal.pgen.1000332)
Supplement: Table S2 — List of primers. (0.12 MB DOC) [file pgen.1000332.s004.doc]

**Table S2:** List of primers

**Plasmid pIgL^GFP2^**

5’-arm forward: GGGGTCGACATATGATACACAGACCTGACATCTC

5’-arm reverse: GGGGGATCCACTAGTATCACATAATCACCAAGGTTGGAAA

3’-arm forward: GGGGGATCCTTCCGCCATGGCCTGGGCTCCTCTC

3’-arm reverse: GGGGCTAGCCCTCTCAGCTTTTTCAGCAGAATAA

Outside primer for target screening: GGGACTAGTAAAATGATGCATAACCTTTTGCACA

Inside primer for target screening: CCCACCGACTCTAGAGGATCATAATCAGCC

**Plasmids pIgL^-,GFP2^, pIgL^W,GFP2^**

5’-arm forward: GGGCTCGAGGGTACTGCGTTTTCCACAAAATTCTCACAG

5’-arm reverse: GGGAGATCTCTGCACTCTGGCACCGTTAAGCACCATCAC

3’-arm forward: GGGTGATCAAGATCTGCTAGCACTAGTGGATCCGTCGA

3’-arm reverse: GGGGAAAAGCGGCCGCCACTGGAAGGAGCTGAAGGCCAC

Outside primer for target screening: AGCTTGGAATTTAACCTCTCCTGTAAA

Inside primer for target screening: CCCACCGACTCTAGAGGATCATAATCAGCC

**Plasmid p+26IgL**^GFP2^

5’-arm forward: GGGATCGATTTCCCTGGTGTGGGTTTTTTTGGGT

5’-arm reverse: GGGATCGATTTGAGAAAGCTCACGCTCCTTCCAA

3’-arm forward: GGGACTAGTCATCTCAGCGGTGCTTATGAATGAC

3’-arm reverse: GGGACTAGTACCCCAAAAAGCAGCCGAGCATTTC

Outside primer for target screening: GCTGCTCACCTTCCTTACCCTGCTGCTCCT

Inside primer for target screening: CCCACCGACTCTAGAGGATCATAATCAGCC

**Plasmid p-15IgL^GFP2^**

5’-arm forward: TTTATCGATGGAGAAGTGAGCGGCAGAGGGAAAT

5’-arm reverse: TTTATCGATTCAGCAGGGCAATGGGCACACACTG

3’-arm forward: TTTACTAGTGCGCAGCAGAGCGCACCAACCACAG

3’-arm reverse: GGGACTAGTTCGACAGCGAGTGCAAAATCATCTT

Outside primer for target screening: GGACCGGGGGTGCTCACAGCACGGCTTT

Inside primer for target screening: CCCACCGACTCTAGAGGATCATAATCAGCC

**Plasmid p+52IgL^GFP2^**

Arms forward: GGGACTAGT TGCCCTTTTGCTTGCAGCCAGCCT

Arms reverse: GGGCTCGAGAATCTCAACAGCTGTGAAGTTTCGA

Outside primer for target screening: GGGGAAACAGTGAGCATGGGGATTCCCT

Inside primer for target screening: CCCACCGACTCTAGAGGATCATAATCAGCC

**Plasmid p-135IgL^GFP2^**

Arms forward: GGGTCTAGAAGTAGTCCAACCAACCTATGCAGT

Arms reverse: GGGCTCGAGTCTGAAGCCTGAAATCACACAGCA

Outside primer for target screening: CTTTCTATCCGTGCTTAGTCTGGTT

Inside primer for target screening: CCCACCGACTCTAGAGGATCATAATCAGCC

**Plasmids pBACH2^GFP2^, pBACH2^W,GFP2^**

5’-arm forward: GGGCTCGAGTAGTCTGTGCATGAAAATGTGCTG

5’-arm reverse: GGGGGATCCAGCAATTAACCAAATCCTCTGACAG

3’-arm forward: GGGGGATCCTCGCTCCTTTATTCTCTCCAGTGGC

3’-arm reverse: GGGTCTAGACAGCCAGCCTATGCACTGCCTCCAC

Outside primer for target screening: GTAACACTGATAAGAGAGAGATCAG

Inside primer for target screening: CCCACCGACTCTAGAGGATCATAATCAGCC

**Plasmids pAID^GFP2^ , pAID^W,GFP2^**

5’-arm forward: GGGCTCGAGGTCATCTGAGAGAGAACCCAGCTGACATGG

5’-arm reverse: GGGGGATCCGCTTCACAACTTAACAGAGGTAGGTTTCA

3’-arm forward: GGGGGATCCGTGAGAGTACTGAACTGAGTCCTGGACAG

3’-arm reverse: GGGACTAGTCAGTCAACATCAGGCAGGAAGATCTGGTTT

Outside primer for target screening: GAGCCTGTGAGGCAACTTCTGTGCAACCCA

Inside primer for target screening: CCCACCGACTCTAGAGGATCATAATCAGCC

**Plasmids pRDM1^GFP2^, pRDM1^W,GFP2^**

5’-arm forward: GAACTCGAGTGCCTGCGGGGAGCGCGCAGACATT

5’-arm reverse: GGGGGATCCTTAGTAGAACTTGATGATGGCATAGCAGCCAG

3’-arm forward: GGGGGATCCTCTGGAGTTTGGCACAGCCACAAGA

3’-arm reverse: GGGGCTAGCCATATGAGGTAGATGTCATTGCACAGCTTT

Outside primer for target screening: GAAAGATCTAGGGGGGCCCGGGCATGGCGGAGGTGTTGG

Inside primer for target screening: CCCACCGACTCTAGAGGATCATAATCAGCC

**Plasmid pRAD52^GFP2^**

5’-arm forward: GGGGAATTCATATCCTCCGCTGTCTCATTGACATACATT

5’-arm reverse: GGGGGATCCTTTGCTGTATATTAATACTTGATGGAAACA

3’-arm forward: GGGGGATCCCAAGCAGTTAATAAGCTTCCACGTCAGATG

3’-arm reverse: GGGTCTAGAGTCGCAGTTTCCGCTGATACGTGGCATCAC

Outside primer for target screening: CTTGCCAAGGGTACAGCTAGCATCCCTCTT

Inside primer for target screening: CCCACCGACTCTAGAGGATCATAATCAGCC

**Plasmid pA-MYB^GFP2^**

5’-arm forward: GGGCTCGAGGATGACTTTCAATATGCAGATCATGACTACG

5’-arm reverse: GGGGGATCCCTCTGATCTTCTTATTTAGTCCAAGG

3’-arm forward: GGGGTCGACGGATCCAATTAAGAAGAAACTGAATGCGTGTTCTTC

3’-arm reverse: GGGGTCGACGCTAGCTCACAGTATCAGAGCCCTTG

Outside primer for target screening: GAGACCGCGCACGAGCGAAGAAGATGATGA

Inside primer for target screening: CCCACCGACTCTAGAGGATCATAATCAGCC

**Plasmid pBCL6^GFP2^**

5’-arm forward: GGGATCGATTCCTTGGCATACGCCCATGGTTTGG
5’-arm reverse: GGGATCGATTCAGGCCATCTTGAGTCTGCCCAGGACG

3’-arm forward: TTTACTAGTGGCAGCCTGAGCTGGTGGGGGCAAG

3’-arm reverse: GGGACTAGTGGAATTCTGAAGAATCATTCTGGTG

Outside primer for target screening: GAGCTGTCTCTGAAAGAAGCGGTGAGAAAA

Inside primer for target screening: CCCACCGACTCTAGAGGATCATAATCAGCC

**Plasmids belonging to “W” fragment deletion series**

5’-arm forward: GGGCTCGAGGGTACTGCGTTTTCCACAAAATTCTCACAG

5’-arm reverse: GGGAGATCTCTGCACTCTGGCACCGTTAAGCACCATCAC

3’-arm forward:GGGTGATCAAGATCTGCTAGCACTAGTGGATCCGTCGA

3’-arm reverse: GGGGAAAAGCGGCCGCCACTGGAAGGAGCTGAAGGCCAC

Outside primer for target screening: AGCTTGGAATTTAACCTCTCCTGTAAA

Inside primer for target screening: CCCACCGACTCTAGAGGATCATAATCAGCC

**Fragment “A”:**

Forward: GAAGCTAGCTTCCGCCATGGCCTGGGCTCCTCTCC

Reverse: GAAACTAGTATTTTTTGACAGCACTTACCTGGACAGCTGAAAAACTGAA

**Fragment “B”:**

Forward: GGGGCTAGCGGTGGATGTGTTTGTTTTACAGAGG

Reverse: GAAGCTAGCGCAAATCTCTGCTAGGGACCTGGCG

**Fragment “C”:**

Forward: GGGGCTAGCGGTGGATGTGTTTGTTTTACAGAGG

Reverse: GAAGCTAGCGTGTGGCAGAGAGTCTACACATGGC

**Fragment “D”:**

Forward: GGGGCTAGCGGTGGATGTGTTTGTTTTACAGAGG

Reverse: GAAGCTAGCATGGAGCTGTACCATGCGGCCTGCT

**Fragment “E”:**

Forward: GGGGCTAGCGGTGGATGTGTTTGTTTTACAGAGG

Reverse: GAAGCTAGCAAGCTCAGGGTCTCAGTTTGGAGCT

**Fragment “F”:**

Forward: GGGGCTAGCGGTGGATGTGTTTGTTTTACAGAGG

Reverse: GAAGCTAGCATTGCTGCAGTGCAAACGCCCTGGT

**Fragment “G”:**

Forward: GGGGCTAGCGGTGGATGTGTTTGTTTTACAGAGG

Reverse: GGGACTAGTTGTTCAGATGGAACTTCTTATGTTC

**Fragment “I”:**

Forward: GGGGCTAGCGGTGGATGTGTTTGTTTTACAGAGG

Reverse: GAAGCTAGCATGGGATGGAAGGGCCCGTCTGGCC

**Fragment “K”:**

Forward: GAAGCTAGCTTTATGCTGGGAACAGGGGGAGTTC

Reverse: GAAGCTAGCATGGGATGGAAGGGCCCGTCTGGCC

**Fragment “L”:**

Forward: GAAGCTAGCAGGACTGTGCTGTCCTCATGCCCCT

Reverse: GAAGCTAGCATGGGATGGAAGGGCCCGTCTGGCC

**Fragment “M”:**

Forward: GAAGCTAGCCACGACAGCTGGGGCCACACAAAGA

Reverse: GAAGCTAGCATGGGATGGAAGGGCCCGTCTGGCC

**Fragment “N”:**

Forward: GAAGCTAGCGTCACAGGTTGTAACAGGCTGACAT

Reverse: GAAGCTAGCATGGGATGGAAGGGCCCGTCTGGCC

**Fragment “P”:**

Forward: GGGGCTAGCTCACAGAAACATTGAAATGGCTCCT

Reverse: GAAGCTAGCATGGGATGGAAGGGCCCGTCTGGCC

**Fragment “S”:**

Forward: GAAGCTAGCTTTATGCTGGGAACAGGGGGAGTTC

Reverse: GAAACTAGTATTGCTGCAGTGCAAACGCCCTGGT

**IRES-Bsr-cassette:**

Forward: AAATGATCACCCCTCTCCCTCCCCCCCCCCTAACGTTACT

Reverse: GGGTGATCAGGATCCGATCCAGACATGATAAGATACATTG

**VJ interveining sequence of unrearranged IgL locus:**

Forward: GGGGGATCCAGATCTGTGACCGGTGCAAGTGATAGAAAACT

Reverse: TACAAAAACCTCCTGCCACTGCAAGGAGCGAGCTGATGGTTTTTACTGTCT

**GFP RT-PCR:**

Forward: GGGTCTAGAGCCATCATGGTGAGCAAGGGCGAGGAGCTGT

Reverse: TGCGGTTCACCAGGGTGTCGCCCTCGAACT

**EF1alpha RT-PCR:**

Forward: GGGAAGCTTCGGAAGAAAGAAGCTAAAGACCATC

Reverse: GGGACTAGTAGAAGAGCGTGCTCACGGGTCTGCC
